# Using the Stable Carbon and Nitrogen Isotope Compositions of Vervet Monkeys (*Chlorocebus pygerythrus*) to Examine Questions in Ethnoprimatology

**DOI:** 10.1371/journal.pone.0100758

**Published:** 2014-07-10

**Authors:** James E. Loudon, J. Paul Grobler, Matt Sponheimer, Kimberly Moyer, Joseph G. Lorenz, Trudy R. Turner

**Affiliations:** 1 Department of Anthropology, East Carolina University, Greenville, North Carolina, United States of America; 2 Department of Anthropology, University of Colorado, Boulder, Boulder, Colorado, United States of America; 3 Department of Genetics, University of the Free State, Bloemfontein, South Africa; 4 University of Colorado, Denver, Denver, Colorado, United States of America; 5 Department of Anthropology, Central Washington University, Ellensburg, Wisconsin, United States of America; 6 Department of Anthropology, University of Wisconsin-Milwaukee, Milwaukee, Wisconisn, United States of America; University of New South Wales, Australia

## Abstract

This study seeks to understand how humans impact the dietary patterns of eight free-ranging vervet monkey (*Chlorocebus pygerythrus*) groups in South Africa using stable isotope analysis. Vervets are omnivores that exploit a wide range of habitats including those that have been anthropogenically-disturbed. As humans encroach upon nonhuman primate landscapes, human-nonhuman primate interconnections become increasingly common, which has led to the rise of the field of ethnoprimatology. To date, many ethnoprimatological studies have examined human-nonhuman primate associations largely in qualitative terms. By using stable carbon (δ^13^C) and nitrogen (δ^15^N) isotope analysis, we use quantitative data to understand the degree to which humans impact vervet monkey dietary patterns. Based on initial behavioral observations we placed the eight groups into three categories of anthropogenic disturbance (low, mid, and high). Using δ^13^C and δ^15^N values we estimated the degree to which each group and each anthropogenically-disturbed category was consuming C_4_ plants (primarily sugar cane, corn, or processed foods incorporating these crops). δ^13^C values were significantly different between groups and categories of anthropogenic-disturbance. δ^15^N values were significantly different at the group level. The two vervet groups with the highest consumption of C_4_ plants inhabited small nature reserves, appeared to interact with humans only sporadically, and were initially placed in the mid level of anthropogenic-disturbance. However, further behavioral observations revealed that the high δ^13^C values exhibited by these groups were linked to previously unseen raiding of C_4_ crops. By revealing these cryptic feeding patterns, this study illustrates the utility of stable isotopes analysis for some ethnoprimatological questions.

## Introduction

This study seeks to understand the impact of anthropogenic disturbance on the dietary ecology of free-ranging South African vervet monkeys (*Chlorocebus pygerythrus*) by analyzing the δ^13^C and δ^15^N values of hair samples. In Africa, the northernmost populations of vervet monkeys range from Senegal and Ethiopia while the southernmost populations live in South Africa [1]. Vervets prefer open habitats including savannas and savanna woodland mosaics but they can also be found inhabiting coastal forest zones [2]–[3]. Their capability to inhabit such a wide breadth of environments is in part due to their ability to subsist on a broad spectrum of foods. Within some portions of their geographic range, vervet monkeys live among humans resulting in human-vervet sympatry. Overlap between humans and monkeys is not limited to vervets, as populations of other gregarious Old World monkeys, most notably Asian macaques and African baboons, regularly live among humans [4]–[6]. Human-nonhuman primate interplays are the focus of the emergent field of ethnoprimatology which employs primatological and cultural anthropological methods to understand human-nonhuman primate interconnections [7]. This interdisciplinary approach allows ethnoprimatologists to address a broad spectrum of anthropological and ecological questions including the types of interactions that occur between humans and nonhuman primates, human-nonhuman primate bi-directional disease transmission, the role of nonhuman primates in belief systems, and the use of nonhuman primates in tourism, entertainment, and research [5]–[6]. Because ethnoprimatologists examine human perceptions and attitudes towards their nonhuman primate associates, ethnoprimatological studies are useful for elucidating the nature of these interconnections by understanding resource overlap and utilization [7]. Knowledge of the human-nonhuman primate conflicts that arise from human encroachment can also provide important insights for conservation efforts [8]–[9]. While ethnoprimatological studies are effective at characterizing human-nonhuman primate relationships, these datasets are largely qualitative employing interviews and questionnaires, but some studies have utilized quantitative techniques including botanical methods to access anthropogenic disturbance and crop damage and behavioral data collection on humans and nonhuman primates [10].

Stable carbon (δ^13^C) and nitrogen (δ^15^N) isotope analysis is a highly quantitative, and an increasingly cost effective method for studying primate diets [11]–[12] with much promise as a supplement to typical ethnoprimatological datasets. Stable isotope studies are based on the principle that “you are what you eat” and the δ^13^C and δ^15^N values of animals are permanently recorded in their tissues and excreta. This allows for the analysis of archived specimens (e.g., museum specimens) to better characterize human and nonhuman primate interactions over longer temporal scales. In the areas of South Africa of principle interest here, most trees, bushes, and shrubs use the C_3_ photosynthetic pathway while most grasses and some sedges use the C_4_ photosynthetic pathway [13]. The carbon isotope compositions of C_3_ and C_4_ plants do not overlap, and stable isotope analysis of a primate’s hair can reveal the relative amount of C_3_ and C_4_ plants it consumed [14]–[15]. As hair grows it also records these contributions through time [16], thus allowing for the detection of switches in diet due to seasonal shifts or migrations [17] or the consumption of new food resources. Vervets with little human interaction eat primarily C_3_ vegetation [18] but in South Africa, C_4_ crops are prevalent (e.g., corn, millet, sorghum and sugarcane) and are consumed by crop raiding vervets. Vervets may also consume human foods that include corn products or C_4_ sugarcane. Thus, δ^13^C values can be used to understand a population’s reliance on C_4_ crops or processed human foods. Nitrogen isotopes can also prove useful as they track trophic level [19] and provide information on an individual’s physiological state and habitat [20]–[21]. They can also track reliance on agricultural products as synthetic and natural fertilizers can highly alter a habitat’s background nitrogen isotope profile [22].

Although stable isotope data have been used to study the diets of modern [23]–[25] and fossil [26]–[28] primates, few studies have used stable isotopes to examine anthropogenic impacts on nonhuman primates. Loudon et al. [29] conducted one such study that combined field observations of ring-tailed lemurs with stable carbon and nitrogen analysis of their hair. The stable isotopes clearly distinguished the lemur groups that utilized human modified landscapes, and demonstrated the potential of stable isotope analysis for addressing questions about anthropogenic disturbance. Schurr et al. [30] conducted a study on the effects of tourist provisioning on the carbon and nitrogen isotope ratios in the hair of Barbary macaques (*Macaca sylvanus*) inhabiting Gibraltar. They found that macaques living in areas most heavily visited by tourists had significantly higher δ^13^C values, probably indicating the consumption of human snack foods which often contain C_4_ sweeteners. The macaques also had lower δ^15^N values, most likely due to consumption of tourist-provided peanuts, legumes with a relatively low nitrogen isotope composition [30].

Given their omnivorous feeding patterns and varying degrees of overlap with humans, vervet monkeys are excellent candidates to examine ethnoprimatological questions using stable isotope data. As elsewhere throughout the continent, free-ranging South African vervet populations primarily inhabit savanna woodlands and riverine forests [31]. Vervets also inhabit anthropogenically-disturbed habitats including farm lands and urban settings and many of the populations which live among humans frequently raid their crops or are provisioned with processed human foods [32]. Here we examine the δ^13^C and δ^15^N values of hair from eight South African groups of vervet monkeys with varying degrees of consumption of human foods and utilization of anthropogenically-disturbed habitats. Estimations of the human impacts were broad and based on preliminary observations. An in-depth study of vervet monkey behavioral ecology is required at each field site to truly understand the nature of these human-vervet monkey interplays, but we can use stable isotope analyses to generate hypotheses regarding each group’s feeding patterns and habitat use. For carbon, we predicted that groups of vervet monkeys with high degrees of human contact and consumption of human foods will exhibit higher δ^13^C values as human foods are frequently processed or sweetened with C_4_ plants (i.e. corn or sugarcane). We also predicted that the vervet groups with high degrees of human contact that regularly consume human foods or raid crops will exhibit lower δ^15^N values as foods grown with synthetic agricultural fertilizers tend to be ^15^N-depleted.

## Methods

### Study Sites

**Figure 1:**
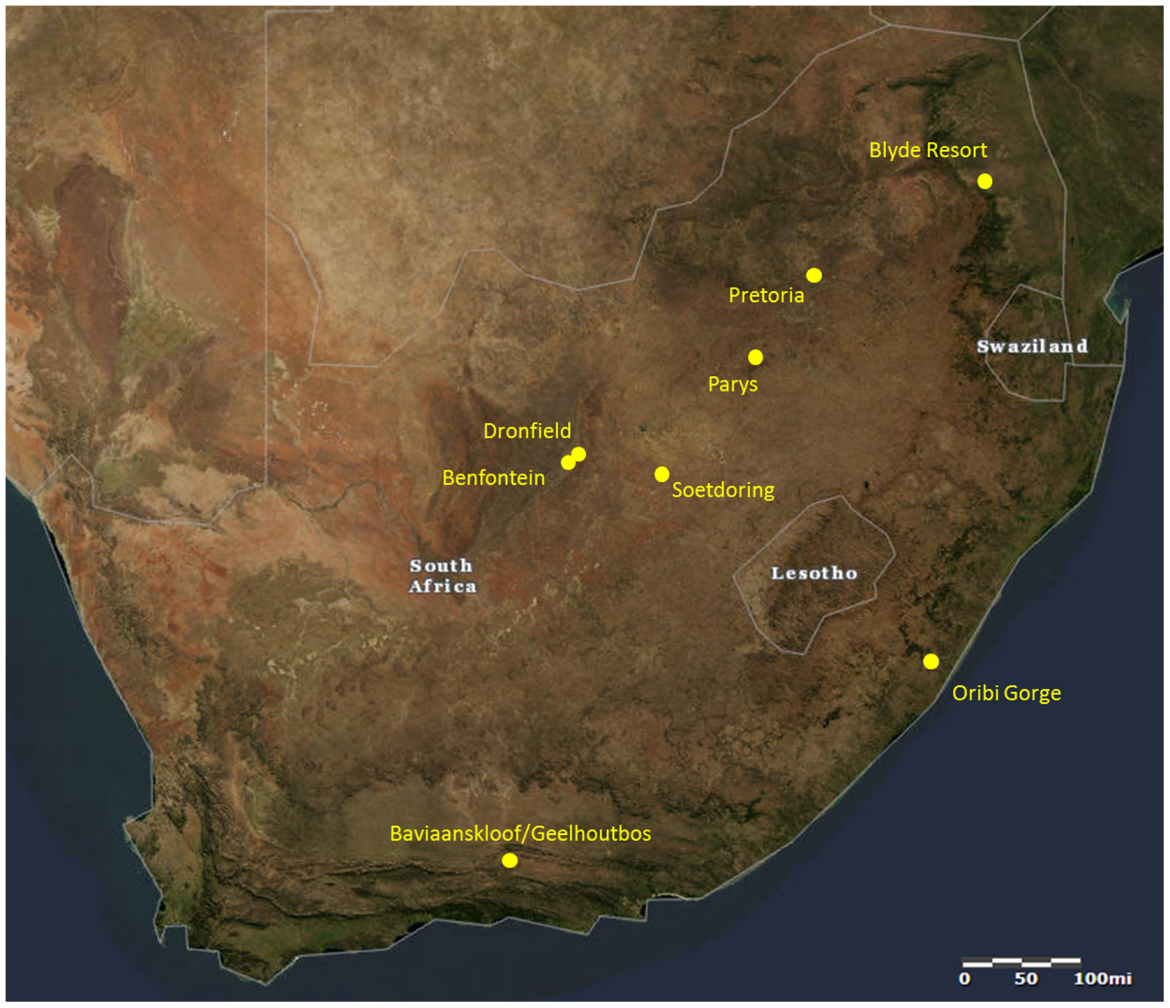
Map of South Africa showing the approximate locations of the eight vervet monkey study groups. Map provided by United States Geological Society National Map Viewer (public domain).

A total of 96 hair samples were collected from eight groups of vervet monkeys (*Chlorocebus pygerythrus*) throughout South Africa (Table 1; Fig. 1). Hair was collected as part of an ongoing study examining vervet monkey population genetics, genomics, phylogeography, and biology [33]–[37]. At each field site, hair was collected from vervet monkeys within their natural range. During hair collection, behavioral observations of the monkeys were conducted by our team and landowners and reserve managers were interviewed to determine the degree of human impact at each site and to note the consumption of human foods by each vervet monkey group [38]. Based on these observations we initially compared the δ^13^C and δ^15^N values of groups living in habitats that were noticeably impacted by humans to groups with little observable anthropogenic disturbance. We then placed each vervet monkey group into one of three categories (low, mid, high) with regard to anthropogenic disturbance. The two groups in the “low” anthropogenic disturbance category (Baviaanskloof/Geelhoutbos and Dronfield) inhabited ranges with little human modification, interacted with humans rarely, and were either rarely or never observed to consume human foods. Three vervet monkey groups that were classified in the “mid” category of anthropogenic disturbance (Benfontein, Oribi Gorge, and Soetdoring) lived in or used habitats modified by humans and were occasionally observed interacting with humans. These groups were observed begging and/or stealing foods from weekend picnic crowds. Thus, sporadic access to human foods supplemented their natural diets. Three vervet monkey groups were categorized as having “high” degrees of anthropogenic disturbance (Blyde Resort, Parys, and Pretoria) because they lived in resorts or city suburbs that were heavily modified by humans and because they consumed large amounts of human foods via provisioning or raiding of discarded human foods.

**Table 1:** Mean and standard deviation for δ^13^C and δ^15^N values and estimated mean and range of the percentage of dietary C_4_ resources consumed by the eight vervet monkey study group organized by level of anthropogenic disturbance.

Study group and number ofmonkeys sampled	Estimated degree ofanthropogenic disturbance	δ^13^C ± SD	δ^15^N ± SD	Estimated % ofdietary C_4_	Range of estimated % ofdietary C_4_
Baviaanskloof/Geelhoutbos (N = 8)	low	−22.5±0.2	6.6±0.7	7.3	5.8–10.1
Dronfield (N = 7)	low	−20.6±0.1	7.1±0.6	21.1	20.1–23.0
**TOTAL (N = 15)**		−**21.6±1.0**	**6.8±0.7**		
Benfontein (N = 5)	mid	−21.4±0.2	10.2±0.4	15.1	12.9–17.3
Oribi Gorge (N = 9)	mid	−17.5±1.5	6.6±0.4	43.1	26.6–56.1
Soetdoring (N = 18)	mid	−19.9±0.4	7.5±0.7	25.8	18.7–29.5
**TOTAL (N = 32)**		−**19.5±1.6**	**7.7±1.3**		
Blyde Resort (N = 9)	high	−20.3±0.8	6.7±0.5	23.1	15.1–33.1
Parys (N = 26)	high	−21.0±0.7	8.2±2.2	18.1	7.9–26.6
Pretoria (N = 14)	high	−20.7±0.4	6.5±0.6	19.8	15.1–23.0
**TOTAL (N = 49)**		−20.8±0.7	**7.4±1.8**		

### Study Subjects, Data Collection, and Ethics Statement

Vervet monkeys were baited with maize and oranges and captured in traps that have been designed to minimize the risk of injury to the monkeys and humans and facilitate the administration of tranquilizers with minimal stress [39]. All techniques for trapping, sedation and sampling were approved by the Interfaculty Animal Ethics Committee of University of the Free State and the Institutional Animal Care and Use Committee at the University of Wisconsin-Milwaukee. Traps were placed in quiet and shaded areas (i.e. under trees) to preclude heat distress within the home range of the trapped monkey. While in the traps, the monkeys were continuously monitored and showed no signs of distress unless approached by humans. Humans approached the traps only to release monkeys. Once sedated, the approximate age and sex of each individual was recorded. Age was determined for subadults by dental eruption sequences. Although the chronological age of adults could not be determined, wear patterns indicated older adults. We also collected body and dental morphometric measurements, and hair was collected from the upper shoulder. The overall physical health of each animal was also accessed and recorded. After examinations, each monkey was safely released near the site of their capture when the effects of their sedation were no longer observable.

### Laboratory and Data Analysis

Hair samples were washed with ethyl alcohol, cut with a razor blade, weighed (∼700 micrograms), and placed in tin capsules. Samples were combusted in an elemental analyzer (Carlo-Erba, Milan, Italy) and analyzed for stable carbon and nitrogen isotope abundances using a flow-through inlet system on a continuous flow isotope ratio mass spectrometer (Finnigan, Bremen, Germany). ^13^C/^12^C and ^15^N/^14^N ratios are expressed using the conventional delta (δ) notation in parts per thousand or permil (‰) relative to the PeeDee Belemite (PDB) and atmospheric N_2_ standards respectively. We estimated the percentage of C_4_ (%C_4_) plant consumption for each group using the following formula from Schwarcz et al. [40]:
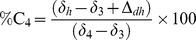



Where δ_h_ is the measured value of the hair sample, Δ_dh_ is the fractionation factor of 3.2 for hair to diet [41], δ_3_ = −26.7‰ (an average value of South African C_3_ plants [42]) and δ_4_ = −12.8‰ (an average value of South African C_4_ plants [42]). To account for unequal variances and/or sample sizes, we used Welch’s analysis of variance (ANOVA) for all statistical analyses comparing groups and the three categories of anthropogenic disturbance using JMP Pro 10.0 and the Games-Howell Test for all pairwise comparisons using Systat 13. We also used generalized linear models with δ^13^C and δ^15^N values as response variables and group, sex, and age as the explanatory variables in JMP Pro 10.0.

## Results

**Figure 2:**
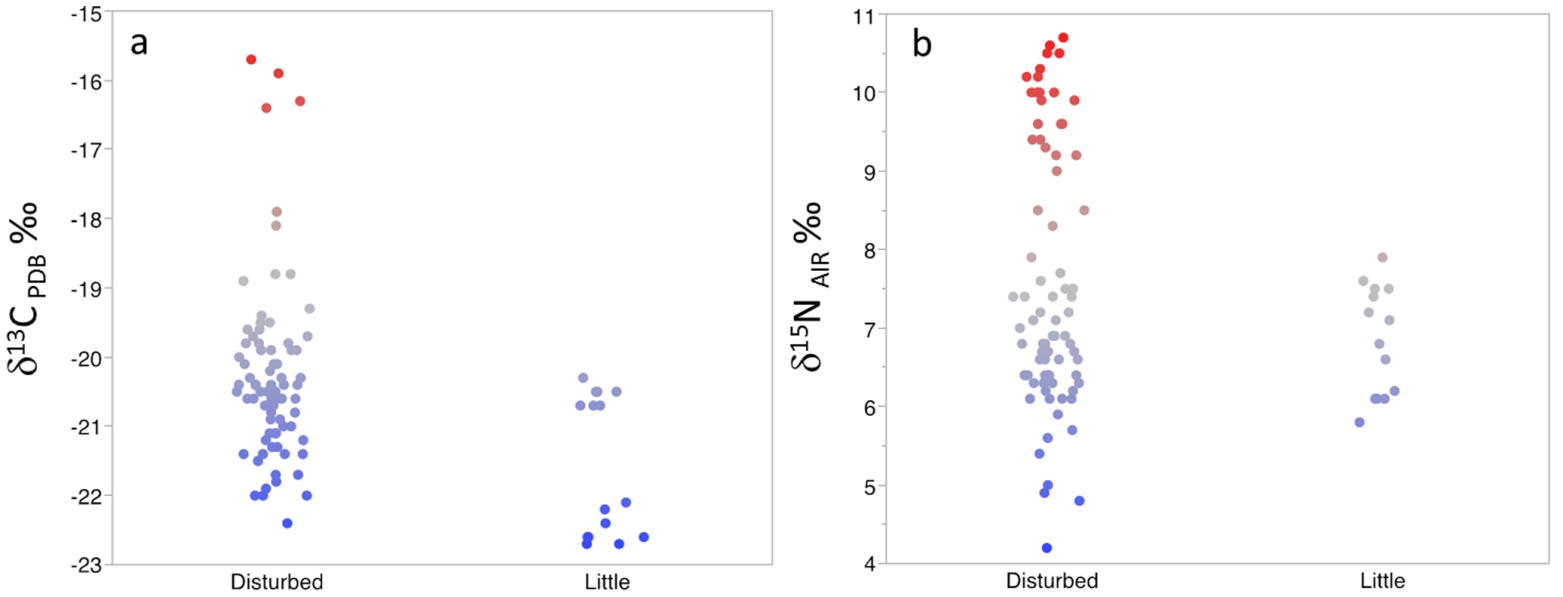
δ^13^C and δ^15^N values for vervet monkey groups living in highly impacted habitats compared to groups with little anthropogenic disturbance. For each figure the lowest values are blue and gradate to the highest values represented by red.

**Figure 3:**
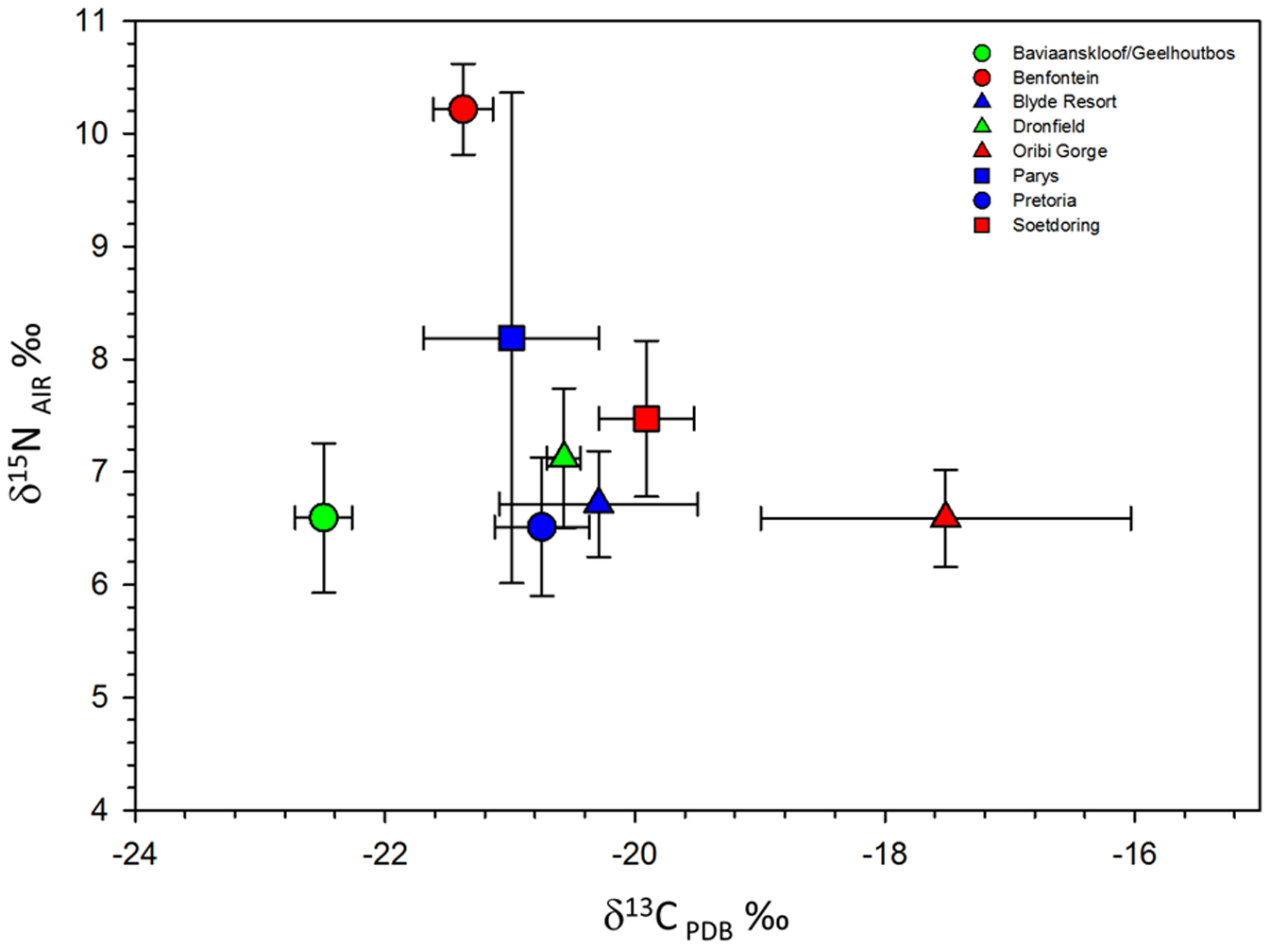
δ^13^C and δ^15^N biplot for each vervet monkey group. Each symbol represents the group mean and bars represent standard deviations. Green symbols represent vervet groups with low levels of human contact, red symbols for levels with mid levels of contact, and blue for those groups with high levels of human contact.

We present all δ^13^C and δ^15^N values in Table S1. The mean and standard deviations of hair δ^13^C and δ^15^N values and the estimated mean and range of C_4_ foods consumed (%C_4_) for each vervet monkey group are shown Table 1. There were significant differences in δ^13^C between the groups living in heavily disturbed habitats to those groups inhabiting environments with little anthropogenic disturbance (F_1,23.2_ = 19.5 P<0.001; Fig. 2a). We also found significant differences in δ^15^N (F 1,50.8 = 7.3 P<0.01; Fig. 2b); however these differences were not significant when we used the Wilcoxon signed-rank test (P = 0.26). Means and standard deviations for the δ^13^C and δ^15^N values for each of the vervet groups are also shown in Fig. 3. Generalized linear models revealed that the effect of group for carbon (P<0.001) and nitrogen (P<0.0001), but not sex (δ^13^C: P = 0.1; δ^15^N: F = 2.22; P = 0.71). For age, there was no effect for carbon (P = 0.09) but we found an effect for nitrogen (P<0.05). Since we found a group effect for both carbon and nitrogen we excluded age and sex as variables in subsequent analyses. There were highly significant differences between the eight vervet monkey groups for δ^13^C (F_7, 28.2_ = 78.9 P<0.0001). The Oribi Gorge group had the highest δ^13^C (−17.5‰ ±1.5; n = 9) while Baviaanskloof/Geelhoutbos was the lowest (−22.5‰ ±0.2; n = 8). Baviaanskloof/Geelhoutbos was significantly different from all groups (Games-Howell P<0.0001) and this vervet group inhabits a region that has abundant C_3_ grasses and very low human impact. Thus, the Baviaanskloof/Geelhoutbos group is close to ideal for having no engagement with C_4_ foods. When the eight groups were placed in categories of low, mid, and high levels of anthropogenic disturbance we found differences in δ^13^C (F_2,31.9_ = 15.8 P<0.0001). Unexpectedly, Oribi Gorge and Soetdoring had the highest mean for estimated percentage of consumed C_4_ foods, even though they were placed in the “mid” level of anthropogenic disturbance based on our initial field observations (Table 1). Pairwise comparisons between the low, mid, and high levels of anthropogenic disturbance were significant between the low and mid level (Games-Howell P<0.0001), mid and high level (Games-Howell P<0.0001), and the low and high comparison (Games-Howell P<0.05). Within group variation was highest at Oribi Gorge (range of 4.1‰) followed by Parys (range of 2.6‰), and Blyde Resort (range of 2.5‰) and lowest among Dronfield (range of 0.4‰), Benfontein (range of 0.6‰), and Baviaanskloof/Geelhoutbos (range of 0.6‰) (see Table S1).

We also found highly significant differences for δ^15^N between the eight vervet groups (F_7,27.9_ = 42.0 P<0.0001). Pretoria had the lowest δ^15^N values (6.5‰ ±0.6, n = 14) while Benfontein (10.2‰ ±0.4, n = 5) was significantly higher than the other sites (Games-Howell P<0.01). There were also differences in δ^15^N values for the three categories of anthropogenic disturbance (F_2,56.1_ = 4.4 P<0.05) and the mid level category was higher than the low category (Games-Howell P<0.05). Within group variation for δ^15^N was highest at Parys (6.4‰), while no other group had a range greater than 2.5‰.

## Discussion

We found significant differences in the δ^13^C values of the eight vervet monkey groups. This was also true when the groups were placed in the categories of anthropogenic disturbance. Differences in δ^13^C values are most likely linked to the consumption of human processed foods enriched with C_4_ plants or the consumption of C_4_ crops. Many of the vervet monkey populations that we studied inhabited ecosystems that include of C_3_, C_4_, and CAM vegetation. However, behavioral observations revealed no consumption of wild C_4_ grasses and succulent CAM plants for any of the eight groups. Our prediction that all groups of vervet monkeys with high degrees of human contact and consumption of human foods would exhibit higher δ^13^C values was not completely supported. For example, the Parys and Pretoria groups both live in urban settings and high levels of C_4_ consumption were expected. C_4_ consumption was ∼18% for Parys and ∼20% for Pretoria, which is considerable and consistent with expectations for monkeys with access to human foods. However, C_4_ consumption for these groups was less than Oribi Gorge (∼43%) and Soetdoring (∼26%) which we placed in the “mid” level of anthropogenic disturbance based on initial behavioral observations (Table 1; Fig. 3). The Oribi Gorge group inhabits a nature reserve with low levels of observed human contact, yet these monkeys exhibited the highest δ^13^C values (−17.5‰ ±1.5‰) indicating substantial consumption of C_4_ resources. These initial results were puzzling; however, we later noted that the Oribi Gorge monkeys were leaving the reserve to raid sugarcane crops located at the reserve’s border. We found a similar scenario for the group that inhabits the Soetdoring Nature Reserve located outside of Bloemfontein. The Soetdoring group interacts only sporadically with tourists which is unlikely to account for its high δ^13^C values (−19.9‰ ±0.4‰) suggesting about 26% of its diet consisted of C_4_ foods (Table 1). However, further behavioral observations of the monkeys revealed that they were swimming across the Modder River that flows within the reserve to raid nearby maize crops. Thus, for some of the groups we studied, isotopes brought to light interactions with human foods that were not initially apparent.

We also found differences in the δ^15^N values of the eight groups as well as between categories of anthropogenic disturbance. Nitrogen isotope values are difficult to interpret since the δ^15^N of soils and plants that are incorporated into animal tissues arise from environmental factors linked to local and global nitrogen cycles and temperature and precipitation [43]. Nonetheless, among the eight groups we studied, Benfontein exhibited the highest δ^15^N values. The Benfontein group is located approximately 20 km from Dronfield but is separated by the city of Kimberley which may be responsible for the differences δ^15^N values (Games-Howell P<0.01) we found. Given the close proximity between the two sites, the differences in the δ^15^N values we detected are likely linked the consumption of human foods as each site is characterized by similar habitats. The Benfontein vervet group occasionally interacted with humans and consumed human foods while the Dronfield vervets rarely interacted with humans (Table 1). More interaction with humans may lead to enrichment in δ^15^N values due to the availability of higher trophic level foods from humans, or the consumption of human foods with ingredients produced in areas with higher δ^15^N values. Among free-ranging Chacma baboons (*Papio ursinus*), higher fecal δ^15^N values were found in animals that were suspected to raid camp sites compared to animals in more remote areas [24]. Moreover, a comparison of three wild ring-tailed lemur (*Lemur catta*) groups revealed that the group that consumed left over human foods at the research camp had the highest hair δ^15^N values [29]. Alternatively, the Benfontein vervets may be relying more heavily on ^15^N enriched insects than the other seven vervet groups and this higher trophic position maybe responsible for the higher δ^15^N values [44].

The intragroup level variability we documented may be the result of individual feeding strategies or related to social rank and access to contested foods. The Oribi Gorge vervet monkeys exhibited the greatest variability in δ^13^C and ranged from −15.7‰ to −19.8‰, followed by the Parys group which ranged from −19.8% to −22.4‰ and the Blyde Resort group that ranged from −18.9‰ to −21.4‰ (Table S1). The Parys vervets exhibited the widest range for δ^15^N which may be an artifact of the sample size (N = 26) or due to differences in diet and/or individual health. Vervets live in groups with differential access to contested resources and highest ranking individuals may have more access to human processed foods with lower δ^15^N values due to artificial fertilizers. Alternatively, differences in δ^15^N may be the result of differences in health. Among free-ranging ring-tailed lemurs, some individuals in poor physical health had the highest δ^15^N values [29]. Low ranking animals with less access to food which are experiencing nutritional stress may also exhibit higher δ^15^N values, a pattern that has been found among birds [20], [45] and humans [46]. The variation we found in δ^15^N values among the Parys group highlights the need for long-term feeding observational data paired with baseline isotope values of the foods that nonhuman primates eat in order to accurately interpret stable isotope values. Our prediction that those vervet monkey groups placed in the high anthropogenic disturbance category (Blyde Resort, Parys, and Pretoria) would have lower δ^15^N values based on the consumption of high levels of human processed foods was not supported. In fact, we found that the groups that were placed in the mid level category of anthropogenic disturbance (Benfontein, Oribi Gorge, and Soetdoring) had the highest δ^15^N values. It is difficult to predict what impact human foods would have on the δ^15^N values we found among these groups since the human foods that were consumed by these vervet groups can be produced at different locations (within South Africa and beyond) potentially driving their δ^15^N values either up or down.

The data presented here suggest that stable isotope analysis can be used for augmenting ethnoprimatological datasets but cannot replace traditional techniques in behavioral observations. Stable isotope values from hair provide feeding data over time and hair collected by ethnoprimatologists can reveal broad dietary patterns before the data collection commences or between field seasons. Stable carbon isotope values from hair reflect the dietary values from foods accrued over days or weeks [16]. Fecal excreta, on the other hand, can be used to analyze the undigested portion of the diet and detect recent dietary switches or feeding patterns. As such, collecting tissues such as hair and excreta with observational feeding data should provide an accurate assessment of feeding patterns and habitat use. Another strength of stable isotope analysis is its ability to reveal cryptic feeding behavior (i.e. crop raiding or consuming human scraps) and assess the degree to which a nocturnal primate is consuming insects or small invertebrates for those ethnoprimatologists observing night-active primates. The study at hand demonstrated the utility of stable isotope analysis for estimating the degree to which humans impact the dietary patterns of free-ranging vervet monkeys by revealing that at least two groups (Oribi Gorge and Soetdoring) were crop raiding C_4_ plants. This is an important find, as vervet monkeys are considered pests in some South African provinces and crop raiding monkeys may be injured or killed. As such, using stable isotope analysis to detect crop raiding by nonhuman primates could be used as an effective tool to develop conservation initiatives and the implementation of deterrents that minimize stress to nonhuman primates. These measures may include the use of fire crackers, water hoses, chemicals that mimic the scent of predators, using sounds to replicate predators, or primate alarm calls [47].

Stable isotope analysis may add to ethnoprimatological studies by confirming or denying people’s perceptions about how local animals are behaving (i.e. consumption of their crops). However, to fully understand people’s attitudes toward the nonhuman primates they live among, conventional ethnoprimatological methods including interviews and questionnaires should not be abandoned. Quantitative and qualitative techniques have their own strengths and weaknesses and combining these methods can be synergistic, resulting in a more appropriate approach to understanding human-nonhuman primate dynamics. It is our hope that combining stable isotope analysis with behavioral observations and ethnographic techniques will not only shed light on human-nonhuman primate interactions in South Africa, but will better develop this technique as a tool for broader ethnoprimatological inquiries specifically related to identifying crop raiding, improving our understanding of nonhuman primate habitat utilization, and documenting anthropogenic effects on nonhuman primate behavioral ecology through time and space.

## Supporting Information

Table S1 δ^13^C and δ^15^N values for each individual as well as their group affiliation and the degree to which the group was impacted by humans.
(DOCX)

Checklist S1 (DOC)
