# Supplementary material for: Using the Stable Carbon and Nitrogen Isotope Compositions of Vervet Monkeys (Chlorocebus pygerythrus) to Examine Questions in Ethnoprimatology
Source: PLoS One. 2014 Jul 10;9(7):e100758. doi: 10.1371/journal.pone.0100758 (PMC4091945; doi:10.1371/journal.pone.0100758)
Supplement: Table S1 — δ13C and δ15N values for each individual as well as their group affiliation and the degree to which the group was impacted by humans. (DOCX) [file pone.0100758.s001.docx]

**Table S1** **δ^13^C and δ^15^N values for each individual as well as their group affiliation and the degree to which the group was impacted by humans.**

| ID | Site | Anthro Input | δ^13^C | δ^15^N |
| --- | --- | --- | --- | --- |
| B 023 | Blyde Resort | high | -21.4 | 7.9 |
| B 029 | Blyde Resort | high | -21.0 | 6.3 |
| B 085 | Blyde Resort | high | -20.7 | 6.3 |
| B 018 | Blyde Resort | high | -20.6 | 6.7 |
| B 019 | Blyde Resort | high | -20.5 | 6.6 |
| B 027 | Blyde Resort | high | -20.5 | 6.7 |
| B 028 | Blyde Resort | high | -19.9 | 6.7 |
| B 031 | Blyde Resort | high | -19.3 | 6.6 |
| B 019 | Blyde Resort | high | -18.9 | 6.7 |
| PA406 | Parys | high | -22.4 | 4.9 |
| PA407 | Parys | high | -22.0 | 5.4 |
| PA408 | Parys | high | -22.0 | 4.2 |
| PA409 | Parys | high | -22.0 | 4.8 |
| PA410 | Parys | high | -21.9 | 5.7 |
| PA411 | Parys | high | -21.8 | 6.1 |
| PA400 | Parys | high | -21.7 | 5.0 |
| PA401 | Parys | high | -21.4 | 6.9 |
| PA402 | Parys | high | -21.3 | 5.6 |
| PA403 | Parys | high | -21.1 | 9.0 |
| PA404 | Parys | high | -21.0 | 9.9 |
| PA405 | Parys | high | -20.9 | 9.6 |
| PA406 | Parys | high | -20.7 | 10.5 |
| PA408 | Parys | high | -20.6 | 9.4 |
| PA409 | Parys | high | -20.6 | 9.9 |
| PA410 | Parys | high | -20.6 | 10.0 |
| PA411 | Parys | high | -20.6 | 10.0 |
| PA412 | Parys | high | -20.6 | 9.4 |
| PA414 | Parys | high | -20.5 | 10.3 |
| PA415 | Parys | high | -20.5 | 10.0 |
| PA416 | Parys | high | -20.5 | 10.6 |
| PA417 | Parys | high | -20.4 | 9.2 |
| PA418 | Parys | high | -20.4 | 8.5 |
| PA419 | Parys | high | -20.3 | 8.3 |
| PA420 | Parys | high | -20.1 | 10.0 |
| PA421 | Parys | high | -19.8 | 9.6 |
| P 068 | Pretoria | high | -21.4 | 6.4 |
| P 068 | Pretoria | high | -21.4 | 6.1 |
| P 071 | Pretoria | high | -21.2 | 6.3 |
| P 074 | Pretoria | high | -21.1 | 6.4 |
| P 070 | Pretoria | high | -20.8 | 6.9 |
| P 061 | Pretoria | high | -20.8 | 6.6 |
| P 062 | Pretoria | high | -20.7 | 6.3 |
| P 063 | Pretoria | high | -20.5 | 8.5 |
| P 064 | Pretoria | high | -20.5 | 6.4 |
| P 067 | Pretoria | high | -20.5 | 6.2 |
| P 078 | Pretoria | high | -20.4 | 6.1 |
| P 079 | Pretoria | high | -20.4 | 6.4 |
| P 066 | Pretoria | high | -20.4 | 6.2 |
| P 060 | Pretoria | high | -20.3 | 6.3 |
| BG486 | Baviaanskloof/Geelhoutbos | low | -22.7 | 6.1 |
| BG487 | Baviaanskloof/Geelhoutbos | low | -22.7 | 6.6 |
| BG488 | Baviaanskloof/Geelhoutbos | low | -22.6 | 7.2 |
| BG489 | Baviaanskloof/Geelhoutbos | low | -22.6 | 5.8 |
| BG496 | Baviaanskloof/Geelhoutbos | low | -22.6 | 6.2 |
| BG497 | Baviaanskloof/Geelhoutbos | low | -22.4 | 6.1 |
| BG498 | Baviaanskloof/Geelhoutbos | low | -22.2 | 7.1 |
| BG485 | Baviaanskloof/Geelhoutbos | low | -22.1 | 7.6 |
| K167 | Dronfield | low | -20.7 | 7.5 |
| K168 | Dronfield | low | -20.7 | 7.5 |
| K169 | Dronfield | low | -20.7 | 6.8 |
| K170 | Dronfield | low | -20.5 | 7.9 |
| K171 | Dronfield | low | -20.5 | 6.8 |
| K172 | Dronfield | low | -20.5 | 7.4 |
| K173 | Dronfield | low | -20.3 | 6.1 |
| K174 | Benfontein | mid | -21.7 | 9.6 |
| K175 | Benfontein | mid | -21.5 | 10.2 |
| K176 | Benfontein | mid | -21.3 | 10.2 |
| K177 | Benfontein | mid | -21.2 | 10.7 |
| K178 | Benfontein | mid | -21.1 | 10.5 |
| O 130 | Oribi Gorge | mid | -19.8 | 6.8 |
| O 131 | Oribi Gorge | mid | -18.8 | 6.9 |
| O 132 | Oribi Gorge | mid | -18.8 | 7.4 |
| O 133 | Oribi Gorge | mid | -18.1 | 6.1 |
| O 134 | Oribi Gorge | mid | -17.9 | 5.9 |
| O 135 | Oribi Gorge | mid | -16.4 | 6.8 |
| O 136 | Oribi Gorge | mid | -16.3 | 6.6 |
| O 137 | Oribi Gorge | mid | -15.9 | 6.4 |
| O 138 | Oribi Gorge | mid | -15.7 | 6.4 |
| SO450 | Soetdoring | mid | -20.9 | 6.8 |
| SO451 | Soetdoring | mid | -20.4 | 6.8 |
| SO452 | Soetdoring | mid | -20.3 | 7.1 |
| SO453 | Soetdoring | mid | -20.2 | 7.5 |
| SO455 | Soetdoring | mid | -20.1 | 7.2 |
| SO456 | Soetdoring | mid | -20.1 | 6.9 |
| SO457 | Soetdoring | mid | -20.0 | 7.0 |
| SO454 | Soetdoring | mid | -19.9 | 7.4 |
| SO459 | Soetdoring | mid | -19.9 | 7.2 |
| SO460 | Soetdoring | mid | -19.9 | 9.2 |
| SO461 | Soetdoring | mid | -19.8 | 7.5 |
| SO462 | Soetdoring | mid | -19.7 | 9.3 |
| SO463 | Soetdoring | mid | -19.7 | 7.1 |
| SO464 | Soetdoring | mid | -19.6 | 7.4 |
| SO465 | Soetdoring | mid | -19.6 | 7.5 |
| SO466 | Soetdoring | mid | -19.5 | 7.6 |
| SO467 | Soetdoring | mid | -19.5 | 7.7 |
| SO468 | Soetdoring | mid | -19.4 | 7.4 |
